# Arrhythmogenic Cardiomyopathy: Definition, Classification and Arrhythmic Risk Stratification

**DOI:** 10.3390/jcm13020456

**Published:** 2024-01-14

**Authors:** Marisa Varrenti, Alberto Preda, Antonio Frontera, Matteo Baroni, Lorenzo Gigli, Sara Vargiu, Giulia Colombo, Marco Carbonaro, Marco Paolucci, Federica Giordano, Fabrizio Guarracini, Patrizio Mazzone

**Affiliations:** Electrophysiology Unit, De Gasperis Cardio Center, Niguarda Hospital, 20162 Milan, Italymarco.carbonaro@ospedaleniguarda.it (M.C.); fabrizio.guarracini@ospedaleniguarda.it (F.G.); patrizio.mazzone@ospedaleniguarda.it (P.M.)

**Keywords:** arrhythmogenic cardiomyopathy, arrhythmic risk, sudden death

## Abstract

Arrhythmogenic cardiomyopathy (ACM) is a heart disease characterized by a fibrotic replacement of myocardial tissue and a consequent predisposition to ventricular arrhythmic events, especially in the young. Post-mortem studies and the subsequent diffusion of cardiac MRI have shown that left ventricular involvement in arrhythmogenic cardiomyopathy is common and often develops early. Regarding the arrhythmic risk stratification, the current scores underestimate the arrhythmic risk of patients with arrhythmogenic cardiomyopathy with left involvement. Indeed, the data on arrhythmic risk stratification in this group of patients are contradictory and not exhaustive, with the consequence of not correctly identifying patients at a high arrhythmic risk who deserve protection from arrhythmic death. We propose a literature review on arrhythmic risk stratification in patients with ACM and left involvement to identify the main features associated with an increased arrhythmic risk in this group of patients.

## 1. Introduction

Arrhythmogenic cardiomyopathy (ACM) is an inherited heart muscle disease with a prevalence of approximately 1:5000 [1], characterized by fibro-fatty myocardial tissue replacement and correlated risk of ventricular arrhythmic events (VA) and sudden cardiac death (SCD), especially in young patients [2]. Originally known as a heart disease affecting only the right ventricle, the subsequent discovery of genetic mutations associated with the disease and the demonstration of disease extension to the left ventricle led to the definition of ‘arrhythmogenic cardiomyopathy’ (ACM) [2]. 

The 2010 task force guidelines defined the criteria for diagnosing right arrhythmogenic cardiomyopathy based exclusively on histopathology data [3]. In 2020, an international consensus document proposed new diagnostic criteria called the “Padua criteria”, introducing criteria for the diagnosis of arrhythmogenic dysplasia with left involvement. Cardiac magnetic resonance imaging (cMRI) is essential for correctly identifying myocardial tissue changes represented by myocardial scarring that characterize this disease phenotype [4].

Regarding the stratification of arrhythmic risk, recent evidence shows how cardiac magnetic resonance imaging (cMRI) and genetic analysis play a crucial role in diagnosing and stratifying the arrhythmic risk of patients with ACM and left ventricular involvement. Still, the literature data on these patients are contradictory and is the risk scores underestimate their incidence of arrhythmic events in the follow-up [5].

We propose a literature review on arrhythmic risk stratification in patients with ACM and left ventricular involvement to identify the main characteristics described in the literature that are associated with an increased arrhythmic risk in this patient group.

## 2. Definition and Classification

A recent international consensus document has provided an update of the criteria for the diagnosis of ACM that includes the three main phenotypic variants, which are arrhythmogenic cardiomyopathy with exclusive right ventricular involvement (ARVC), arrhythmogenic cardiomyopathy with exclusive left ventricular involvement (ALVC) and arrhythmogenic cardiomyopathy with biventricular involvement (ABVC). With a multidisciplinary approach that includes the analysis of morphofunctional and structural abnormalities, emphasizing the importance of the use of cardiac magnetic resonance imaging, in addition to electrocardiographic abnormalities to the analysis of ventricular arrhythmias and family and genetic history, the criteria for a correct diagnosis are defined [6]. Clinically, ventricular arrhythmias and sudden cardiac death (SCD) may be the first manifestation of the disease, which can progress to biventricular heart failure and the need for cardiac transplantation [1]. In 2020, Bariani and colleagues described the “hot phases” disease in patients with ACM, which is an acute phase of disease characterized by chest pain, release of myocardionecrosis enzymes, and presence of electrocardiographic changes in the absence of coronary artery disease. The pathogenetic mechanism is unclear; however, it seems that the inflammatory process may be a trigger for subsequent tissue necrosis and replacement with fibroadipose tissue. In a clinical setting that mimics myocarditis, a family history of ACM and positive genetics should support the differential diagnosis [7].

Most recently, the Padua group proposed a new definition: “scarring/arrhythmogenic cardiomyopathy” (S/ACM), where not the localization but the presence of myocardial scarring define this cardiomyopathy [8]. 

Myocardial scarring is typical of these cardiomyopathies and is the principal cause of the mainly arrhythmic clinical manifestations regardless of disease etiology [9]. The latest ESC guidelines on managing cardiomyopathies recommend an approach to classification and diagnosis based on the predominant phenotype at the time of presentation [10]. The main change of this classification is the identification of a group defined as non-dilated left ventricular cardiomyopathy (NDLVC), which is characterized by the presence or absence of systolic dysfunction and the presence of non-ischemic scarring or adipose replacement of the left ventricle independent of wall motion abnormalities, or left ventricular hypokinesis in the absence of scarring. 

## 3. Left Ventricular Involvement in Arrhythmogenic Cardiomyopathy

Initially, the classic phenotype of arrhythmogenic right ventricular heart disease was described by early and predominant involvement of the right ventricle in the absence or only slight involvement of the left ventricle, which was regarded as a progression of the disease itself. Subsequent studies, especially since the introduction of cardiac magnetic resonance imaging, have shown that left-sided involvement in arrhythmogenic cardiomyopathy is common and often early [11].

A histopathological study shows that, in patients diagnosed with ACM, the left ventricle was involved in 76% of cases with a prevalent subepicardial extension of the left ventricular free wall. Phenotypic features of left ACM included low-amplitude QRS complexes in limb leads, T-wave inversion in lateral or inferolateral leads, ventricular arrhythmias with right bundle branch block (RBBB) morphology denoting LV origin, non-dilated left ventricle with normal or mildly depressed left ventricular function and evidence of LGE on cardiac MRI with non-ischemic extension [12,13].

Different studies report a higher prevalence of left ventricular involvement in patients with ARVC [14].

In their experience, Chen and colleagues describe how out of 68 patients with ARCV only 40% had preserved left ventricular EF, while 60% had a left ventricular EF less than 55%. Of these, all patients had a cardiac MRI with left ventricular LGE [15]. 

A different study reported similar findings with left ventricular dysfunction and left ventricular LGE in 38% of patients with ARCV [16]. In the Italian study by Cipriani and colleagues on 87 patients with ARCV, 47% had LVEF ≤50% and LGE LV, while 19% showed LV LGE but their LVEF was >50%. Furthermore, the group with left ventricular dysfunction or the presence of LV LGE showed a more significant prevalence of DSP mutations without showing differences in terms of right ventricular dysfunction or proper LGE involvement [11]. 

In another study, out of 220 cases of ARCV, 24% had left ventricular dysfunction at CMR, most commonly associated with carriers of mutations in the desmocollin-2, phospholamban and desmoplakin genes [17]. 

In their study, Akdis et al. report their experience with ARCVs enrolled in the Swiss registry: Of 64 patients enrolled, 26% had no left ventricular involvement at baseline evaluation but the same occurred during follow-up. The presence of left ventricular involvement at baseline evaluation was more often associated with the desmoplakin gene mutation [18].

In Aquaro’s study on 140 patients, only 7% had left ventricular dysfunction and considered LVEF to be < 50%. However, 48.5% of patients had LV involvement, defined as the presence of LV LGE (35%), LV wall motion abnormalities (21%), LV fat infiltration (31%), LV dilatation (6%) or left ventricular dysfunction (7%) [19].

Numerous clinical cases described in the literature report whether left arrhythmogenic cardiomyopathy is associated with left ventricular dysfunction and genetic mutations [20,21,22,23,24,25,26,27,28,29,30,31]. 

## 4. Genetics

In accordance with the recent consensus document proposing a revision of the diagnostic criteria for arrhythmogenic cardiomyopathy, in an etiological classification, we distinguish idiopathic ACM, non-genetically related ACM (mainly inflammatory) and genetically determined ACM. In this last group, we distinguish defects in desmosomal and non-desmosomal genes. Defects in desmosomal genes include mutations in Desmoplakine (DSP), Plakoglobin (JUP), Desmocollin (DCS) and PlakophilinC (PKP2). 

Possible genes coding for non-desmosomal genes include mutations in Phospholamban (PLN), Filamin C (FLNC), Desmin (DES), Lamin A/C (LMNA) as well as other proteins involved in musculoskeletal tissue diseases [6].

Several mutations in the desmoplakin gene are linked to ACM, which is characteristically manifested by early involvement of the left ventricle and mutations in PLN are associated with altered intracellular calcium flow with increased susceptibility to arrhythmic events. Mutations in FLNC also have a phenotype overlapping that of DCM [12].

Genotype definition is not only important for diagnosis but also for prognostic stratification. As indicated in the latest European Society of Cardiology guidelines on Cardiomyopathies, specific genetic mutations are associated with a higher arrhythmic risk [10]. Indeed, several authors have raised the importance of genetics in the correct stratification of arrhythmic risk and the construction of the relevant risk scores (see Section 5).

## 5. Arrhythmic Risk Stratification

Sudden cardiac death in ACM may be the first presentation of the disease. Data show that overall mortality in patients with ACM is less than 1% up to a maximum of 3.6% in high-level centers following high-risk patients [32].

The latest guidelines of the European Society of Cardiology on the primary prevention of sudden cardiac death (SCD) considers ICD implantation in patients with definite ARVC and arrhythmic syncope (Class of Recommendations IIa—B), severe left ventricular dysfunction (LVEF ≤ 35%) and right ventricular dysfunction (Recommendations IIa—C). Still, according to the authors, an ICD should be considered in symptomatic ARVC patients with moderate RV and or LV dysfunction and NSVT inducible to PES [33]. Notably, these guidelines do not consider the role of cardiac magnetic resonance imaging and the presence and/or extent of myocardial fibrosis in arrhythmic risk stratification. 

The European guidelines on the management of cardiomyopathies highlight that there are no randomized trials on the utilization of ICDs in patients with NDLVC and mild or moderately impaired EF. Literature data show that genetics is crucial for proper arrhythmic risk stratification. The guidelines consider it reasonable to consider ICD implantation in patients with a family history of sudden cardiac death, a history of even non-sustained ventricular arrhythmias and the presence of significant LGE on cardiac MRI [10].

In 2019, Cadrin-Tourigny and colleagues proposed a risk score to predict arrhythmic events in patients with ARVC. This study was based on 528 patients with ARVC, according to the 2010 Task Force criteria, who had no previous history of ventricular arrhythmias or sudden cardiac death [5]. The patients were followed for 4.83 years, and, using a Cox model, eight potential predictors of arrhythmic events in the follow-up were identified: history of cardiac syncope, sex, age, TVNS, number of premature ventricular complexes (PVC) in 24 h, T-wave inversion lead, and right ventricular ejection fraction (RVEF) and left ventricular ejection fraction (LVEF) [5]. Three years later, Jordan et al. validated this risk score in a cohort of 429 ARVC patients [34], even if a further study showed this score to be more effective among patients with a positive genetic mutation (especially in the PKP2 subgroup) but with limited usefulness in patients with an elusive genetic mutation, suggesting that genotype should be included in a risk model [35]. However, this score may underestimate the incidence of arrhythmic events in patients with ARVC and left-sided involvement [19]. 

In their systematic literature review, Bosman et al. analyzed predictors of ventricular arrhythmias or sudden cardiac death in patients with ARVC in 45 studies with a median follow-up of 5.0 years. The *mean rate of observed arrhythmic events* was 10.6%/year in definite ARVC patients, 10.0%/year in borderline disease and 3.7%/year in patients with genetic mutations. In patients with definite ARVC, the consistently predictive risk factors were sex, history of syncope, T-wave inversion in the V3 lead, RV dysfunction and history of (non)sustained VT/VF. In patients with borderline ARVC, two additional predictive factors were identified: inducibility during electrophysiological study and strenuous exercise. Moreover, in mutation carriers, all of the predictors mentioned above—plus ventricular ectopy, multiple ARVC-related pathogenic mutations, left ventricular dysfunction and palpitations/presyncope—determined arrhythmic risk. In conclusion, the predictors of arrhythmic events in patients with ARVC were the male gender, history of syncope, T-wave inversion in more than three leads, right ventricular dysfunction and a previous history of ventricular arrhythmic events (sustained or not) [36]. The 2019 consensus document recommends arrhythmic risk stratification in patients with ARVC and ACM, based on the presence of ventricular arrhythmias (sustained and non-sustained), the severity of ventricular dysfunction and the presence or absence of specific genetic mutations. There is no consideration of the role of cardiac magnetic resonance imaging and, therefore, the presence and extent of late gadolinium enhancement in arrhythmic risk stratification [37].

According to the largest cohort of ARVC patients without history of sustained ventricular arrhythmias (VA) at diagnosis, the score for SCD risk stratification was proposed (the 2019 ARVC risk score, www.arvcrisk.com) [5]. The risk score that proved to predict the risk of major arrhythmic events in this group of patients included the following variables: sex, age, history of syncope within the last 6 months, history of non-sustained ventricular tachycardia (NSVT), number of PVCs/24 h, extent of T-wave inversion in the anterior and inferior leads, and ventricular ejection fraction

The 2019 ARVC risk score showed to be a strong predictor of risk in patients with the PKP2 pathogenic variant, but not in patients with the DSP pathogenic variant and in a gene-elusive cohort [38,39].

Regarding arrhythmic risk stratification in patients with arrhythmogenic heart disease and left involvement, there are discordant data in the literature. In their retrospective study, Zghaib et al. report that out of 73 patients with ARVD/C, 51% had left involvement with evidence of fibrosis and adipose infiltration, mainly located in the apical-lateral region of the left ventricle and with the subendocardial distribution. The left ventricular LGE was related with the development of ventricular arrhythmias at follow-up only on univariate analysis. In contrast, on multivariate analysis only the history of sustained ventricular arrhythmias and right dysfunction were related to the presence of ventricular arrhythmias during follow-up [40]. In their multicenter study, Corrado et al. analyzed data from 132 patients diagnosed with ARVC who received an ICD implantation, showed that both cardiac arrest and ventricular tachycardia with hemodynamic impairment, decreased age and decreased left ventricular ejection fraction were predictors of major arrhythmic events [41].

Hulot and colleagues showed that in a group of 130 patients with ARVD and a follow-up of 8.1 years, predictors of long-term cardiovascular death were a history of ventricular tachycardia, right ventricular failure and left ventricular dysfunction [42].

In their study, Aquaro et al. demonstrated that left involvement in patients with ARCV is associated with a worse prognosis than right involvement alone. Furthermore, the ARCV risk score can predict events in patients with right involvement but underestimate the risk in patients with left ventricular involvement. In multivariate analysis, ARVC with an LV-dominant presentation and the 5-year ARVC risk score were independent predictors of major cardiac events. The other evidence was the significant negative predictive value of cardiac MRI. Indeed, there was no evidence of events in patients with a definite diagnosis of ARVC [19].

One of the most significant reported experiences in patients diagnosed with ARVD and receiving ICDs in secondary prevention comes from the American registry. In a cohort of 312 patients with a median follow-up of 9 years, 60% of patients had an appropriate ICD intervention after a median time of about 7 years. History of VTS, arrhythmias inducible to PES, male gender, T-wave inversions and PVC/24 h were predictors of arrhythmic events with appropriate ICD interventions.

After a literature research through PubMed using the keywords “ARVC and left ventricular involvement”, we chose a selected number of studies that reported the characteristics of patients with ARVC and left ventricular involvement, as shown in Table 1 [17,43,44,45,46,47,48,49,50,51,52,53].

Left ventricular involvement in patients with ACM is reported in up to 87% of cases, whether extensive or focal, and numerous studies show it to be a negative prognostic predictor [55].

Peters and colleagues had already shown in 1999 that left ventricular involvement was a predictor of arrhythmic events in patients with right ventricular dysplasia [56]. Moreover, in their work, Turrini and colleagues reported a history of syncope, left and right ventricular involvement and QRS dispersion as predictors of cardiac death [57].

Some years later, Maupain et al. reported the results of their analysis of 137 patients with ARVC/D showing that left involvement in terms of EF ≤ to 50% was an independent predictor of ventricular arrhythmic events [58].

In their meta-analysis of 26 studies involving a total of 2680 patients with an average follow-up of 5.4 years, Bazoukis et al. identified left ventricular dysfunction as one of the possible predictors of arrhythmic events in the follow-up along with other factors such as the male gender and a history of syncope [59].

The 2015 consensus document identified three categories of arrhythmic risk for patients with ARVC: low, moderate and high. Patients considered to be at ‘high risk’ followed the classic indications of ICD implantation in secondary prevention after a major arrhythmic event or in the case of severe ventricular dysfunction. In contrast, patients were defined as ‘moderate risk’ when major risk factors such as a previous history of syncope, non-sustained left ventricular tachycardia and moderate right and/or left ventricular dysfunction were present. ICD implantation as primary prevention was indicated in this group of patients when one or more of the arrhythmic risk factors were present [60]. As reported in the work of Aquaro and colleagues, patients with ARVC with dominant left ventricular and/or biventricular presentation had worse event-free survival than patients with ARVC and right involvement only. In contrast, there was no difference in survival in patients with ARVC with only right involvement and those with negative cMRI. Interestingly, patients with negative MRI had no events at follow-up which demonstrates the high negative predictive power and the importance of phenotypic expression in arrhythmic risk stratification of patients with ARVC. Also, in the same paper, the authors suggest that the arrhythmic risk in patients with ARVC and left dominance is higher even in the absence of obvious functional or structural impairment, reinforcing the role of MRI in the prognostic stratification of these patients who may have preserved left ventricular function [19].

As reported in the guidelines on the prevention of sudden cardiac [33] death, the identification of ARVC patients with proper risk of sudden cardiac death is not easy. 

Predictive factors include arrhythmic syncope and left and right ventricular dysfunction. ICD should be considered in patients with severe RV and/or LV dysfunction. According to experts, ICD should be considered in symptomatic patients with moderate RV and/or LV dysfunction and NSVT or SMVT inducible to PES [33].

The same indications are reported in recent guidelines on cardiomyopathies regarding arrhythmic risk stratification in patients with ARVC [10].

Regarding patients with ARVC and left involvement according to the new classification of the latest ESC guidelines [10], these are included in the group of patients with non-dilated left ventricular cardiomyopathy (NDLVC). Regarding ICD implantation in primary prevention, no randomized clinical trials are available that analyze the usefulness of the ICD in preventing sudden cardiac death in patients with mild or moderate dysfunction. The guidelines emphasize the importance of genetics in the stratification of arrhythmic risk and identify genes associated with high arrhythmic risk regardless of LVEF:PLN, TMEM43, DES, DSP, LMNA, FLNC (truncating variants) and RBM20. Whenever possible, the use of dedicated risk scores in ICD indication is recommended (https://lmna-risk-vta.fr for LMNA and https://plnriskcalculator.shinyapps.io/final_shiny for PLN p.Arg14del) [10].

Moreover, the guidelines suggest that ICD implantation should be considered as a primary prevention method in patients with NSVT, a family history of SCD or significant LGE at MRI.

In accordance with the current definition of ACM, extended LGE refers to the ‘ring-like’ pattern, i.e., LGE involving more than three segments of the left ventricle in a short axis section on cardiac MRI [6]. In addition, it should be considered that the left ventricular LGE > 15% on cardiac MRI has been proven to be a strong predictor of arrhythmic events at follow-up in patients with ACM [61].

The literature data prove how left ventricular involvement plays a role in the prognosis of patients with right ventricular arrhythmogenic heart disease. Despite this, there is currently no score that can accurately predict the arrhythmic risk for these patients. 

## 6. Role of Cardiac MRIs

The introduction of cardiac MRIs in clinical practice has played a fundamental role in the correct identification and diagnosis of patients with ACM, especially for the forms with left and biventricular involvement [62,63]. Its role also seems to be fundamental in the prognostic stratification of patients with ACM.

Aquaro and colleagues demonstrated that left involvement in ACM is a strong independent predictor of major arrhythmic events in patients with ARVC [19,64].

In their study, Zhang and colleagues showed that the presence of left ventricular LGE > 15% in patients with ACM was a strong predictor of arrhythmic events [61]. See Table 2.

There is also evidence that the left involvement displayed using cardiac MRI is associated with an increased likelihood of heart failure events in patients with ACM [65].

## 7. Conclusions

**Figure 1:**
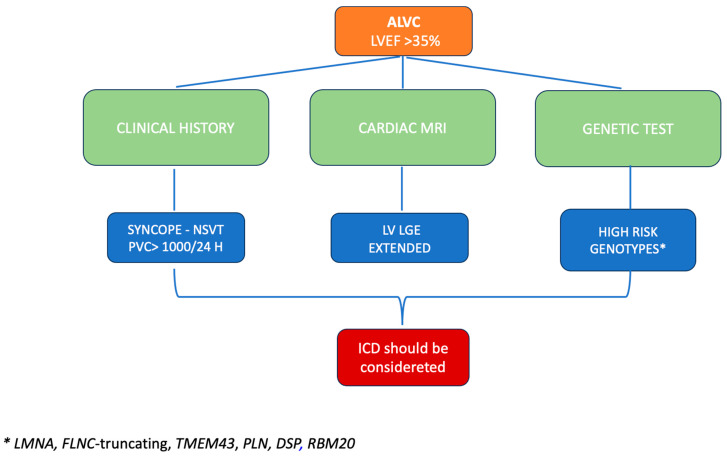
In patients with arrhythmogenic heart disease with left-sided involvement, arrhythmic risk stratification should go beyond the ejection fraction by considering the patient’s clinical history, the genetic mutations associated with increased arrhythmic risk and the presence of left ventricular LGE displayed using cardiac MRI. ALVC, arrhythmogenic left ventricular cardiomyopathy; ICD, implantable cardioverter defibrillator; LVEF, left ventricular ejection fraction; NSVT, non-sustained ventricular tachycardia; LV, left ventricular; LGE, late gadolinium enhancement.

In our opinion, by analyzing the data available in the literature and considering the latest international guidelines, two factors are present in the stratification of the arrhythmic risk of patients with ARVC and left involvement, which are the presence of genetic mutations associated with an increased arrhythmic risk and the presence of late gadolinium enhancement at MRI. However, there is still no quantification of the degree of fibrosis in accurately defining the arrhythmic risk of these patients, and each case must be analyzed individually (See Figure 1).

Also, in patients with preserved or mildly reduced EF, risk factors for major arrhythmic events should be carefully evaluated. The indication for an ICD in primary prevention should be considered in patients with mildly reduced or preserved EF who present prognostic factors related to the development of major arrhythmic events: a previous history of syncope, the presence of non-sustained arrhythmic events or high 24 h ventricular extrasystole burden, together with data on genetics and the presence of late gadolinium enhancement on cardiac MRI. The latter two should be mandatorily performed in all patients with suspected cardiomyopathy.

In conclusion, left ventricular involvement in arrhythmogenic cardiomyopathy increases the arrhythmic risk, which should go beyond the ejection fraction, considering not only the patient’s clinical history, but the genetic mutations and the presence of myocardial scarring displayed using cardiac MRI.

## Figures and Tables

**Table 1:** ARVC and left involvement. Literature data.

Author	Year	Patients (n°)	Left Ventricular Involvement (n°, %)	Phenotypic Characteristics of Patients with Left Involvement
Cipriani et al. [11]	2020	87	58 (67%)	Low QRS voltages in limb leads. T-wave inversion in the inferolateral leads. major ventricular arrhythmias.
Zghaib et al. [40]	2021	73	37 (51%)	Proband. Lower RVEF. Lower LVEF. Higher right ventricular wall motion abnormality.
Aquaro et al. [19]	2020	140	68 (48%)	Lower RVEF. Lower LVEF. Lack of PKP2 mutation. Presence of DSG2 mutation.
Shen et al. [16]	2019	60	35 (58%)	Lower LVEF.
López-Moreno et al. [54]	2016	30	18 (60%)	Higher LVEDD. Higher degree of RV and LV dysfunction.
Akdis et al. [18]	2020	64	29 (61%)	Lower RVEF. Lower LVEF. Higher HF symptoms.

ARVC, arrhythmogenic right ventricular cardiomyopathy; RVEF, right ventricular ejection fraction; LVEF, left ventricular ejection fraction; LV, left ventricular; HF, heart failure; LVEDD, left ventricular end-diastolic diameter; PKP2, Plakophilin C; DSG2, Desmoglein 2.

**Table 2:** Left ventricular involvement as a predictor of arrhythmic events in ARVD/C patients. Literature data.

Author	Year	Patients, n°	Follow-Up	Predictors of Arrhythmic Events
Peters et al. [56]	1999	121	-	Left ventricular involvement. Right ventricular dilatation.
Turrini et al. [57]	2003	60	-	Left ventricular involvement. Syncope. QRS dispersion.
Corrado et al. [41]	2003	132	39 ± 25 months	LVEF. Age. Cardiac arrest. VT with hemodynamic compromise.
Hulot et al. [42]	2004	130	8.1 ± 7.8 years	Left ventricular dysfunction. Right ventricular failure.
Schuler et al. [51]	2012	26	10 years	Left ventricular involvement. Age
Bhonsale et al. [17]	2015	577	7 years	Left ventricular dysfunction. Male sex. Multiple genetic mutations.
Maupain et al. [58]	2018	137	42 ± 31 months	LVEF ≤ 50%. EPS positive. Physical activity.
Aquaro et al. [64]	2018	175	1558 days	Left ventricular involvement.
Bazoukis [59]	2019	2680	5.4 years	Left ventricular dysfunction.
Julia Cadrin-Tourigny et al. [5]	2019	528	4.8 years	Age. Sex. Cardiac syncope. Non-sustained ventricular tachycardia. Number of PVC/24 h. Number of leads with T-wave inversion. Right ventricular ejection fractions. Left ventricular ejection fractions.
Aquaro et al. [19]	2020	140	5 years	LV involvement. Five-year ARVC risk score.
Zhang et al. [61]	2021	88	4 years	LV-LGE at cardiac MRI.

ARVC, arrhythmogenic right ventricular cardiomyopathy; LVEF, left ventricular ejection fraction; LV, left ventricular; LGE, late gadolinium enhancement; VT, ventricular tachycardia; EPS: electrophysiology study; PVC: premature ventricular complex; MRI: magnetic resonance imaging.
